# Oral food desensitisation: are the patients safe after the end of protocol?

**DOI:** 10.1186/2045-7022-5-S3-P152

**Published:** 2015-03-30

**Authors:** Ana Paula Castro Moschione, Andrea Keiko Fujinami Gushken, Glauce Hiromi Yonamine, Cleonir Lui Beck, Antonio Carlos Pastorino, Mayra Dorna, Fabio Morato-Castro, Cristina Miuki Jacob

**Affiliations:** 1 Faculty of Medicine, University of Sao Paulo, Sao Paulo, Brazil

## 

Desensitization protocols are increasing in food allergy centers and a higher number of patients submitted to these protocols have been showing a higher number of adverse events even after well succeed procedure. The aim of this study is to describe adverse reactions after cow's milk desensitization.

## Methods

13 patients (3M: 10F, median age=11.8; 5.9-16.6) were submitted to a protocol that includes twice in a week increasing milk dose with initial dose calculated through an end point test. Adverse reactions were described according "Severity of Clinical Symptoms" by Sampson.

## Results

Before desensitization protocol all patients had presented anaphylaxis, being 9 in the last year, regarding accidental ingestion. Mean serum level of specific IgE to milk and casein were 55.24 and 47.3, respectively. From all well succeeded patients (200ml of milk at the end of protocol,) 9 patients still presented isolated reactions, being 3 Grade 1, 1 Grade 2, 0 grade 3, 5 Grade 4, 0 Grade 5. Only in 2 patients it was possible to detect a risk factor associated, that was infection, NSAIs, fasting and exercise, alcohol. In 2 patients the symptoms decreased after fractioning milk ingestion.

## Conclusion

Although desensitization procedures are a hope for a new quality of life in these patients, more studies must be developed to guarantee a really safe future for these people.

